# Editorial: Targeting the PD-1/PD-L1 Cancer Immune Evasion Axis: Challenges and Emerging Strategies

**DOI:** 10.3389/fphar.2020.591188

**Published:** 2020-09-25

**Authors:** Yiting Wang, Hubing Shi, Huan Meng, Jie Xu

**Affiliations:** ^1^Institutes of Biomedical Sciences, Zhongshan-Xuhui Hospital, Key Laboratory of Epigenetics and Metabolism, Fudan University, Shanghai, China; ^2^Renji Hospital, School of Medicine, Shanghai Jiao Tong University, Shanghai, China; ^3^The State Key Laboratory of Biotherapy, Sichuan University West China Hospital, Chengdu, China; ^4^Division of Nanomedicine, Department of Medicine, University of California, Los Angeles, Los Angeles, CA, United States

**Keywords:** immune checkpoint, cancer therapy, gene regulation, acquired resistance, CAR-T and CAR-NK cell-therapy

Extensive exploration and utilization of cancer immunotherapy have revealed promising but challenging prospect of this field. The clinical benefits of Immune Checkpoint Blockade Therapy (ICBT) were limited due to intrinsic and adaptive resistance as well as emerging side effects. In this field, existing translational and basic investigations remain limited and controversial, revealing our insufficient understanding of cancer immune evasion mechanisms. This topic includes 16 updated articles. They focus on various aspects, including but not limited to analysis of clinical significance, side effects of ICBT, regulation of immune checkpoints and novel strategies.

The prognostic role of PD-L1 expression in immunotherapy was proposed before (Patel and Kurzrock, 2015), but the correlation between PD-L1 expression and prognosis are not well addressed in many cancer types. Under this topic, the prognostic and clinicopathological significance of PD-L1 expression were analyzed in colorectal cancer, prostate cancer, and bladder cancer in three articles respectively. By systematically reviewing and meta-analyzing past studies, they all concluded that PD-L1 expression is associated with poor prognosis. But they differ from each other on focuses according to characteristics of different cancer types. In colorectal cancer (CRC), PD-1/PD-L1 axis has been widely acknowledged as a promising therapeutic target, supported by recent clinical trials. This study not only evaluated the prognostic significance of PD-L1 expression, but also suggested that PD-L1 expression might be used as a biomarker for prognosis. In addition, the association between PD-L1 expression and location and differentiation of CRC, among other clinicopathological parameters, were statistically significant according to this analysis. Authors proposed several possible mechanisms that could upregulate PD-L1 level, such as Microsatellite Instability (MSI) (Li, He et al.). In prostate cancer, PD-L1 DNA methylation (mPD-L1) was additionally analyzed. The risk of biochemical recurrence was significantly higher in patients with higher mPD-L1. PD-L1 was specially analyzed with several clinical parameters, among which Gleason score and androgen receptor status were found significantly related to PD-L1. This study presented credible analysis, though credibility of results is somehow less convincing due to limited number of studies (Li, Huang et al.). In Bladder cancer, PD-L1 expression was found significantly correlated with tumor stage and metastasis, in addition to poor prognosis (Zhu et al.). These three articles mentioned above summarized eligible studies to help us obtain a better understanding of the role of PD-L1 expression in multiple cancers. They also suggested that more work should be done to deal with existing controversies, both clinically and mechanically.

Owing to massive application and experiments of immunotherapy, more and more clinicians are confronted with immune-related adverse effects (irAEs) of immunotherapies. These adverse effects largely cut down on the benefits patients can achieve from immunotherapy (Martins et al., 2019). There are four articles here that discuss unfavorable effects, such as diabetes, immune-related pneumonitis, and liver fibrosis. Xiuju Liang and colleagues reported a case and provided a literature review on immune-related pneumonitis. The patient was given an additional PD-1 inhibitor after her disease progressed on previous PD-L1 inhibitor. After that she rapidly developed a severe steroid-resistant pneumonitis, suggesting that clinicians should take a history of pneumonitis into consideration as a possible risk factor for immune-related pneumonitis (Liang et al.). Lijun Da and colleagues conducted a meta-analysis on randomized controlled trials about organ-specific irAEs. They involved 8 RCTs with 2716 patients and listed the most common adverse effects of Immune Checkpoint Inhibitors (ICI). Colitis was ranked as most common irAE, followed by hypothyroidism, hepatitis, hypophysitis, hyperthyroidism, and pneumonitis. Notably, ICI combination therapy significantly increased the risk of all irAEs mentioned above (Da et al.), which supplemented the former case report about pneumonitis and provided with solid evidence. These two articles highlighted the risk of combining ICIs, which deserves more attention and investigations. Jingli Lu and colleagues presented a meta-analysis of 40 randomized controlled trials and conclude that the risk od new-onset diabetes with ICI is rather low but unneglectable, appealing more studies to substantiate these findings (Lu et al.). Clinical use of PD-L1 can also be combined with inhibition of transforming growth factor-β (TGF-β), which displayed additive antitumor response in a sub group of cancer patients. Xiutao Fu and colleages digged into the underlying mechanism of miR-20a-5p/TGFBR2 axis that dominantly regulates TGF-β pathway. Results suggested that miR-20a-5p plays a critical role in liver fibrosis through pro-inflammatory macrophages (Fu et al.).

The mechanisms underlying tumor immune evasion, though popularly investigated, are still poorly understood. Prognostic factors that may contribute to adverse reactions and efficacy are reviewed and discussed by Xinyu Yan and colleagues. Their summary categorized the contributing factors into four group, the characteristics of tumor, the features of microenvironment, the factors in peripheral blood and the individuality of host, illustrating a comprehensive frame of tumor-host interaction network (Yan et al.). The efficacy of ICBT, often disrupted by adaptive and intrinsic drug resistance, is a major concern about the application of PD-1/PD-L1 inhibition therapy. Luisa Chocarro de Erauso and colleagues attempted to find out predictive biomarkers to stratify patients with probability of response to ICBT by clarifying the molecular mechanism of PD-1/PD-L1 ICBT resistance (Chocarro de Erauso et al.). Peixin Dong and Oliviero Marinelli both put their focus on gynecological malignancies. Dong and colleagues emphasized the importance of acknowledging tumor-intrinsic signaling of PD-L1 in modulating immune-independent functions such as epithelial-to-mesenchymal transition (EMT), cancer stem cell (CSC)-like phenotype, metastasis and drug resistance. They carried on a meta-analysis that demonstrated coamplification between PD-L1 and MYC, SOX2, N-cadherin and SNAI1. Their findings may evoke more researches on related pathways and the role of PD-L1 (Dong et al.). On the other hand, Marinelli and colleagues summarized the controversial role of PD-L1 as a prognostic factor in gynecological malignancies, while stressed the importance of a novel molecule, PD-L2, in improving efficiency of immunotherapy (Marinelli et al.). Recent studies of post-translational modification of PD-L1 have broaden the horizon of PD-L1 pathway regulation (Wang et al., 2019; Yao et al., 2019). A summary of multifaceted regulation of PD-L1 is composed by Yiting Wang, providing a variety of routes that may be promising targets for new therapies (Wang et al.). The tumor immune microenvironment (TIME) is widely acknowledged as a pivotal factor contributing to tumor immune evasion, but the complexity and individual differences vastly hold back the understanding and utilization of it. Weilun Fu and colleagues leveraged mass cytometry with a panel of 33 markers to analyze the infiltrating immune cells in diffuse astrocytoma and oligodendroglioma. The composition and status of immune cells were assessed. This article provides a methodology of analyzing tumor-immune interaction, by directly profiling the landscape of TIME (Fu et al.). This method may be applied in more researches to unveil the features and mechanisms of cancer immunology.

Optimistically, novel strategies are constantly emerging. Abscopal effects (AbE) was discovered 60 years ago. It refers to systematic antitumor reactions caused by radiation therapy (RT), which leads to regression of nonirradiated lesions (NiLs). Accumulating evidence fostered a growing consensus that combination of immunotherapy and RT provides a better opportunity to boost AbE (Ngwa et al., 2018). Trommer and colleagues conducted a retrospective study on patients with metastatic cancer. With strict inclusion criteria, they concluded that combination of RT and ICI provided stronger AbE, compared to ICI alone (Trommer et al.). Their results encouragingly call on more prospective researches on this topic to provide solid and sophisticated guidelines on combination of ICI and RT. The unprecedented breakthrough brought by chimeric antigen receptor-redirected T (CAR T) cell therapy marked a new mile stone of cancer immunotherapy. Disruption of endogenous inhibitory immune checkpoints on T cells presents additive immune response. Xingliang Guo and colleagues used the CRISPR/Cas9 gene-editing system to knock down the PD-1 expression on the Glypican-3 (GPC3)-targeted second-generation CAR T cells employing CD28 as the costimulatory domain. *In vitro*, CAR T cells were cocultured with PD-L1 expressing Hepatocellular carcinoma (HCC). PD-1 disrupted GPC3-CAR T cells displayed not only stronger CAR-dependent antitumor activity but also less sign of exhaustion, compared to wild-type GPC3-CAR T cells. *In vivo*, PD-1 disrupted GPC3-CAR T cells showed improved persistence and infiltration in subcutaneous xenograft tumor model of NSG mice (Guo et al.). Discovery of eligible new targets is another strategy to tackle with the dilemma in immunotherapy. B7H3, also known as CD276, is an immune checkpoint molecule that is aberrantly over-expressed in many types of cancer. Peixin Dong and colleagues reviewed its role in modulating cancer behavior in many aspects and employed miRNA as potential therapeutic strategy (Dong et al.).

Under this topic we have seen analysis of prognostic role of PD-L1 expression in various cancer types, which requires more mechanistical investigations to turn the phenomenon into deep-scale understanding and translational strategies. Researches on the adverse effects of ICIs quantified the frequency of common irAEs. Specially, combination of different ICIs significantly increases risk of adverse effects, which deserves to be emphasized and considered in clinical scenes. Mechanisms underlying the modulation of PD-1/PD-L1 axis are explored and summarized, hoping to deepen and widen the understanding on PD-L1 and its role in cancer immune evasion, progression as well as resistance to ICIs. Novel strategies including combination of therapies, disruption of checkpoints on CAR T cells and employment of new targets provides promising and encouraging methodologies. Discussion and exploration on the cancer immune evasion and immune checkpoint targeting therapy will continue to provide exciting findings and benefit patients.

## Author Contributions

YW, HS, HM, and JX wrote the manuscript.

## Funding

This work was supported by National Key R & D Program of China (2016YFC0906002, 2016YFC0906002), National Natural Science Foundation of China (No: 82030104, 81874050, 81572326), and Basic Research Projects of Shanghai Science and Technology Innovation Action Plan (20JC1410700).

## Conflict of Interest

The authors declare that the research was conducted in the absence of any commercial or financial relationships that could be construed as a potential conflict of interest.
